# Slowing down too fast

**DOI:** 10.1007/s12471-020-01522-x

**Published:** 2020-12-01

**Authors:** U. C. Nguyên, P. M. J. C. Kuijpers

**Affiliations:** Department of Cardiology, Maastricht University Medical Center, Maastricht, The Netherlands

**Fig. 1:**
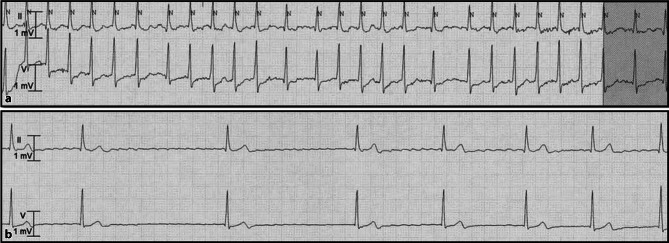
Telemetric recordings during hospitalisation. **a** Atrial fibrillation with a fast ventricular rate. **b** Bradycardic episode after administration of metoprolol

A 77-year-old man was hospitalised for non-ST-elevation myocardial infarction. His telemetry recording showed atrial fibrillation with a fast ventricular rate (Fig. 1a). The patient was normotensive and was treated with metoprolol tartrate 25 mg three times daily to control the heart rate and angina. Briefly after administration of the first metoprolol dose, he became hypotensive (RR 80/60 mm Hg) and bradycardic (Fig. 1b). He was placed in Trendelenburg position, after which his symptoms decreased substantially. Although his blood pressure normalised after metoprolol discontinuation, the bradycardia persisted for over two days.

Cytochrome P450 (CYP) polymorphism analyses were carried out and revealed the patient was a very poor (*4/*4) metaboliser of CYP2D6 and CYP2C9; CYP2C19 function was diminished. The symptomatic hypotension and bradycardia after metoprolol were attributed to the CYP2D6 polymorphism [1, 2]. Genetic polymorphism testing of CYP enzymes may therefore be considered, or should even be advised, in patients who demonstrate strong side effects to beta-blockers and in patients with polypharmacy.
